# The Effect of Cell Culture Passage on the Efficacy of Mesenchymal Stromal Cells as a Cell Therapy Treatment

**DOI:** 10.3390/jcm13092480

**Published:** 2024-04-24

**Authors:** MDolores Carmona-Luque, Antonio Ballesteros-Ribelles, Alejandro Millán-López, Alfonso Blanco, Sonia Nogueras, Concha Herrera

**Affiliations:** 1 Cell Therapy Group, Maimonides Institute of Biomedical Research in Cordoba (IMIBIC), 14004 Cordoba, Spain; antonio.ballesteros@imibic.org (A.B.-R.); alejandro.millan@imibic.org (A.M.-L.); inmaculada.herrera.sspa@juntadeandalucia.es (C.H.); 2 Anatomy and Comparative Pathology Department, University of Cordoba, 14014 Cordoba, Spain; 3 Department of Hematology, Reina Sofia University Hospital, University of Cordoba, 14014 Cordoba, Spain

**Keywords:** bone marrow-derived mesenchymal stromal cell, adipose tissue-derived mesenchymal stromal cells, cell therapy, cardiac regeneration, immunophenotype, ultrastructure, differentiation, cytokine secretion

## Abstract

**Background/Objective:** Mesenchymal Stromal Cells (MSCs) have been considered a promising treatment for several diseases, such as cardiac injuries. Many studies have analyzed their functional properties; however, few studies have characterized MSCs through successive culture passages. The main objective of this work was to analyze the phenotype and functionality of MSCs isolated from two different sources in five culture passages to determine if the culture passage might influence the efficacy of MSCs as a cell therapy treatment. **Methods:** Bone Marrow (BM)-MSCs were harvested from the femur of Wistar rats (n = 17) and Adipose Tissue(AT)-MSCs were isolated from inguinal fat (n = 17). MSCs were cultured for five culture passages, and the immunophenotype was analyzed by flow cytometry, the functionality was characterized by adipogenic, osteogenic, and chondrogenic differentiation assays, and cytokine secretion capacity was determined through the quantification of the Vascular Endothelial Growth-Factor, Fibroblast Growth-Factor2, and Transforming Growth-Factorβ1 in the cell supernatant. The ultrastructure of MSCs was analyzed by transmission electron microscopy. **Results:** BM-MSCs exhibited typical phenotypes in culture passages two, four, and five, and their differentiation capacity showed an irregular profile throughout the five culture passages analyzed. AT-MSCs showed a normal phenotype and differentiation capacity in all the culture passages. BM- and AT-MSCs did not modify their secretion ability or ultrastructural morphology. **Conclusions:** Throughout the culture passages, BM-MSCs, but not AT-MSCs, exhibited changes in their functional and phenotypic characteristic that might affect their efficacy as a cell therapy treatment. Therefore, the culture passage selected should be considered for the application of MSCs as a cell therapy treatment.

## 1. Introduction

In the early 1970s, Friedenstein et al. described the existence of multipotent mesenchymal cells in bone marrow (BM) isolated from mice with the ability to form colonies and differentiate into adipocytes, chondrocytes, and osteoblasts [1]. Twenty years later, Caplan established their name as Mesenchymal Stem Cells [2]. Since then, numerous studies have been carried out using MSCs as a cell therapy treatment applied for the treatment of different diseases. According to the guidelines of the International Society of Cell and Gene Therapy [3], human MSCs and those derived from different mammals, including murine, are characterized by the expression of a specific panel of positive markers on the cell surface such as CD105 (or endoglin), CD73, and CD90, and negative markers such as CD45, CD34, CD14 (or CD11), CD97-α (or CD19), and HLA-DR. Moreover, MSCs must show the plastic adhesion property under standard culture conditions, and they must have the in vitro ability to differentiate at least to the adipogenic, osteogenic, and chondrogenic lineages. These criteria have been established as the main criteria that an MSC must meet to be defined as such.

MSCs secrete different chemokines, cytokines, and extracellular matrix proteins involved in various biological processes including hematopoiesis, angiogenesis, leukocyte trafficking, and immune and inflammatory responses [4]. Moreover, MSCs have a non-immunogenic profile because they present the Major Histocompatibility Complex (MHC) type I but lack the MHC type II. This property leads to immunosuppressive properties such as the inactivation of T cells [5]. Additionally, MSCs exhibit an additional immunomodulatory effect on immune system cells when they are in an inflammatory microenvironment. It has been shown that MSCs are capable of generating an immunomodulatory effect on the adaptive immune response through the reduction of certain markers in certain cells of the immune system, thus favoring the modulation of this immune response, which is very useful in certain applications of Regenerative Medicine and especially in allogeneic transplantation [6]. Considering these biological characteristics, MSCs have been considered a promising cell source for the treatment of autoimmune, inflammatory, and degenerative diseases.

Previous pre-clinical and clinical studies have shown that MSCs are present in many tissues such as BM, adipose tissue (AT), umbilical cord blood, Wharton’s jelly, skin, hair follicles, dental pulp, endometrium, amniotic fluid, fetal liver, placenta, synovium and synovial fluid, mobilized peripheral blood, and muscle [7]. To date, bone marrow-derived MSCs (BM-MSCs) and adipose tissue-derived MSCs (AT-MSCs) represent the optimal stem cell sources for tissue engineering and regenerative medicine [8]. Besides this, both cell types have been the main sources of MSCs used in preclinical and clinical studies related to wound healing and repairing [9,10], the nerve system [11,12], autoimmune disorders [13], cartilage and bone regeneration [14,15], immune and hematological disorders [16], and cardiovascular and muscle diseases [17].

In this scenario, many studies have compared both cell types; however, most of them analyzed MSCs expanded in the same culture passage. They compared their functional properties, such as expansion potential, immunophenotype, differentiation capacity, cytokine secretion ability, and senescence [18,19,20], in a single culture passage, and their results showed functional differences concerning their differentiation and proliferation capacities [18,19], and their secretome [20]. They concluded that specific protocols should be considered for each cell type to improve the results derived from its application in clinical practice.

To date, few published reports have analyzed the functional properties of MSCs derived from BM and AT in successive culture passages [21,22,23]; however, in the conclusion derived from these few studies published, there is no consensus regarding the number of culture passages that must be selected for the clinical or preclinical application of BM-MSCs and AT-MSCs.

Therefore, the present work has aimed to characterize rat-derived BM-MSCs and AT-MSCs expanded in five different culture passages (from culture passage 1 to culture passage 5) and analyze their immunophenotype, functionality, and ultrastructural morphology over different culture passages. To carry out this study, we selected the five culture passages most used in stem cell-based therapies [9,10,11,12,13,14,15,16,17].

## 2. Materials and Methods

### 2.1. Isolation and Culture of BM-MSCs and AT-MSCs

To develop this study, an extensive phenotypic and functional characterization and an ultrastructural analysis were performed on both cell types included: bone marrow-derived MSCs and adipose tissue-derived MSCs, throughout five culture passages.

MSCs were harvested from pathogen-free-9-week-old male Wistar rats. Animals were obtained from the Maimonides Institute for Biomedical Research (IMIBIC, Cordoba, Spain) and housed in an air-conditioned room with light–dark cycles and ad libitum food and drink. Animal experiments were approved by the Institutional Animal Care and Use Committee of Cordoba University and developed under European (86/609/EEC, 24 November 1986) and Spanish (DOG 214/1997, of July 30) regulations for animal stabling, handling, experimentation, and other scientific purposes. All animals were treated humanely by the Guidelines for the Care and Use of Laboratory Animals. At the end of all procedures, animals were anesthetized by intraperitoneal (IP) injection of thiopental (50 mg/kg) and euthanized by exsanguination.

BM was harvested from 17 rats by flushing femurs and tibiae marrow cavities with phosphate-buffered saline (PBS) (Gibco, Grand Island, NY, USA). The cell suspension was filtered, transferred to a sterile tube containing PBS with penicillin (100 U/mL) and streptomycin (100 µg/mL), and washed by centrifugation. The final cell suspension was resuspended in α-minimal essential medium (α-MEM) (Lonza, Verviers, Belgium) enriched with 15% fetal bovine serum (FBS) (Gibco, Waltham, MA, USA), 100 U/mL penicillin, 100 µg/mL streptomycin, 2 mM ultra-glutamine (Lonza, Verviers, Belgium), and 1 ng/mL rat Fibroblast Growth Factor-2 (rFGF-2) (Sigma-Aldrich, St. Louis, MO, USA) [24,25].

Subcutaneous AT was extracted from the inguinal fat of 17 rats and transferred to a sterile tube containing PBS with penicillin (100 U/mL) and streptomycin (100 µg/mL). AT was minced and digested in a collagenase type I solution (Sigma-Aldrich, St. Louis, MO, USA) under constant agitation at 37 °C, washed with FBS to neutralize collagenase action, and filtered. The final cell suspension was resuspended in α-MEM supplemented with FBS, antibiotics, ultra-glutamine, and rFGF-2.

BM- and AT-derived cell suspensions were seeded in 25 cm^2^ and 75 cm^2^ culture flasks (Nunc™, Thermo Scientific, Roskilde, Denmark), respectively, and incubated at 37 °C in a humidified atmosphere containing 5% CO_2_. Non-adherent cells were removed after 3 days, the culture medium was refreshed at 2–3 day intervals, and adherent cells were harvested by trypsinization (Trypsin-EDTA, Sigma-Aldrich, St. Louis, MO, USA) when they reached 80% confluence to be reseeded in a new flask. This initial passage of the primary cell culture was referred to as passage 0. From this, the procedure was replicated to obtain MSCs in culture passages 1, 2, 3, 4, and 5.

### 2.2. Phenotypic Characterization

BM-MSCs (n = 17) and AT-MSCs (n = 17) at culture passages 1, 2, 3, 4, and 5 were phenotypically characterized by flow cytometry using mouse monoclonal antibodies anti-rat CD34 (Santa Cruz, Biotechnology, Dallas, TX, USA), CD45, and CD90 (BD Pharmigen™, San Diego, CA, USA) and hamster monoclonal antibody anti-rat CD29 (BD Pharmigen™, San Diego, CA, USA).

The acceptance criteria for MSCs establish that CD90 and CD29 markers must represent more than 95% of the cell population whilst CD34 and CD45 markers are less than 5% [3]. The forward (FSC) and side (SSC) scatter of cells was also determined. Cells were acquired on a MACSQuant flow cytometer (Miltenyi-Biotech, Bergisch Gladbach, UK) and analyzed using the MACSQuantify™ software, Version 2.5. At least 100.000 events were analyzed for each marker.

### 2.3. Functional Characterization

BM-MSCs (n = 17) and AT-MSCs (n = 17) at culture passages 1, 2, 3, 4, and 5 were functionally characterized by analyzing their adipogenic, osteogenic, and chondrogenic differentiation capacity.

#### 2.3.1. Adipogenic Differentiation Assay

To determine their adipogenic potential, MSCs were seeded at 2.9 × 10^5^ cells/cm^2^ in a 6-well plate and cultured in completed α-MEM until the cells reached confluence. Then, the basal medium was replaced by an adipogenic induction medium supplemented with specific SingleQuots^®^ Kits (Lonza, Verviers, Belgium). Cells were cultured for ten days more. Finally, fat vacuoles derived from the differentiated MSCs were fixed and stained with Oil Red-O solution (Sigma-Aldrich, St. Louis, MO, USA). Stained cells were observed using a 10× objective from a Nikon Eclipse TE2000-S inverted microscope (Nikon, Boston, MA, USA). Ten digitalized images were acquired per sample with a Nikon DS-U2 camera and analyzed with NIS-Element software, version 3.2, to determine MSCs adipogenic differentiation capacity.

#### 2.3.2. Osteogenic Differentiation Assay

To determine osteogenic potential, MSCs were seeded at 1.9 × 10^5^ cells/cm^2^ in a 6-well plate and cultured with osteogenic induction medium supplemented with specific SingleQuots^®^ Kits (Lonza, Verviers, Belgium). The culture medium was refreshed twice a week. After 3 weeks, the mineralization of differentiated MSCs was observed in the bottom of the wells. Cells were fixed, and the mineralization was stained with Alizarin Red S solution (Sigma-Aldrich, St. Louis, MO, USA) and observed using a 10× objective from a Nikon Eclipse TE2000-S inverted microscope (Nikon, Boston, Massachusetts, USA). Ten digitalized images were acquired per sample with a Nikon DS-U2 camera and analyzed with NIS-Element version 3.2 software, to determine MSCs’ osteogenic differentiation capacity.

#### 2.3.3. Chondrogenic Differentiation Assay

For chondrogenic differentiation, a micromass culture system was used. According to the manufacturer’s instructions (StemXVivo Rat Chondrogenic Differentiation R&D System), 2.5 × 10^5^ MSCs resuspended in 500 µL of completed chondrogenic differentiation media (R&D System, Minneapolis, MN, USA) were seeded in a 15 mL conical tube (BD Falcon™, Franklin Lakes, NJ, USA). Cells were incubated for 21 days at 37 °C in a humidified atmosphere containing 5% CO_2_ and a specific medium which was refreshed twice a week. Finally, a differentiated micromass derived from differentiated MSCs was observed in the bottom of the tube. This micromass was fixed, embedded in epoxy resin, and stained with uranyl acetate and lead citrate to identify its morphology at the ultrastructural level. This analysis was performed with a Jeol Jem 1400 (Jeol, Akishima, Tokyo, Japan) transmission electron microscope (TEM) located in the Central Support Service for Research (SCAI) of the Cordoba University (Cordoba, Spain) [26]. Digitalized images were acquired with the microanalysis software Inca Energy 200 Tem and Aztec. Ten images were acquired and analyzed per sample.

### 2.4. Cytokines Quantification in Cell Culture Supernatants

On culture day 0, BM-MSCs (n = 17) and AT-MSCs (n = 17) at passages 1, 2, 3, 4, and 5 were resuspended in 2 mL of completed α-MEM, seeded at 6.6 × 10^3^ cells/cm^2^ in a 6-well plate per triplicate and incubated at 37 °C in a humidified atmosphere containing 5% CO_2_. Cell culture supernatants were collected after seven days. To express the final cytokine concentration as cytokine culture concentration (pg/mL) secreted per 10^4^ cells in 24 h, the day before supernatant collection, on day 6, the culture medium was refreshed. Finally, supernatant samples were preserved at −80 °C until their quantification. Vascular Endothelial Growth Factor (VEGF), Fibroblast Growth Factor-2 (FGF-2), and Transforming Growth Factor-β1 (TGF-β1) concentrations were quantified by ELISA kits (RayBiotech^®^, Norcross, GA, USA, R&D System Minneapolis MN and Invitrogen™, Carlsbad, CA, USA, respectively) according to the manufacturer’s instructions.

### 2.5. Ultrastructural Analysis by Transmission Electron Microscopy

BM-MSC and AT-MSC morphologies at culture passages 1, 2, 3, 4, and 5 were analyzed by TEM at the ultrastructural level. For this analysis, MSCs were expanded to the five analyzed culture passages, trypsinized, washed, and centrifugated to form a cell pellet at the bottom of the centrifuge tube. The pellets were fixed in 2.5% glutaraldehyde solution overnight and a secondary fixation was carried out in osmium tetroxide. Fixed pellets were embedded in epoxy resin, and ultra-thin sections (0.1 µm) were cut with an ultra-microtome LKB III and stained with uranyl acetate and lead citrate. The ultrastructural morphology of the MSCs was analyzed in a Jeol Jem 1400 (Jeol, Akishima, Tokyo, Japan) TEM located in the SCAI of the Cordoba University (Cordoba, Spain). All sections analyzed were carried out with digitalized images acquired in .tiff mode using the microanalysis software Inca Energy 200 Tem and Aztec. Twenty images were acquired and analyzed per sample.

### 2.6. Statistical Analysis

All data were expressed as Mean ± Standard Deviation (SD) unless otherwise indicated. Nonparametric statistical analysis was used (n < 30) according to the results derived from the Shapiro–Wilk analysis. Comparisons between all groups were made with the Kruskal–Wallis test and those between 2 groups were made with the Mann–Whitney U test. Intra-individual comparison of baseline versus follow-up was analyzed by a nonparametric Wilcoxon test. Statistical significance was accepted when the *p*-value was ≤0.05. All analyses were performed with PASW Statistic 18 (IBM SPSS, Armonk, NY, USA) software.

## 3. Results

### 3.1. Phenotypic Characterization

The results of surface markers quantification are shown in Table 1.

Phenotypic characterization of BM-MSCs proved no expression of the hematopoietic markers (CD34, CD45). More than 90% of MSCs derived from BM expressed the typical MSCs marker proteins CD90 and CD29 in culture passages 2, 4, and 5. The CD90 surface marker was detected at a percentage less than 90% in cells expanded in passages 1 and 3.

Concerning the FSC and SSC analysis performed in BM-MSCs, no significant differences were observed between all culture passages (*p* = ns).

Regarding the AT-MSCs phenotypic characterization, it was observed that the percentage of cells that expressed the hematopoietic markers (CD34, CD45) was less than 1%, and more than 90% of cells expressed the surface proteins CD90 and CD29 in all culture passages. These cell populations showed no significant differences between the FSC and SSC results (*p* = ns).

### 3.2. Functional Characterization

Functional characterization performed in the five culture passages of BM-MSCs and AT-MSCs was evaluated by analyzing their adipogenic, osteogenic, and chondrogenic differentiation capacities.

**Figure 1:**
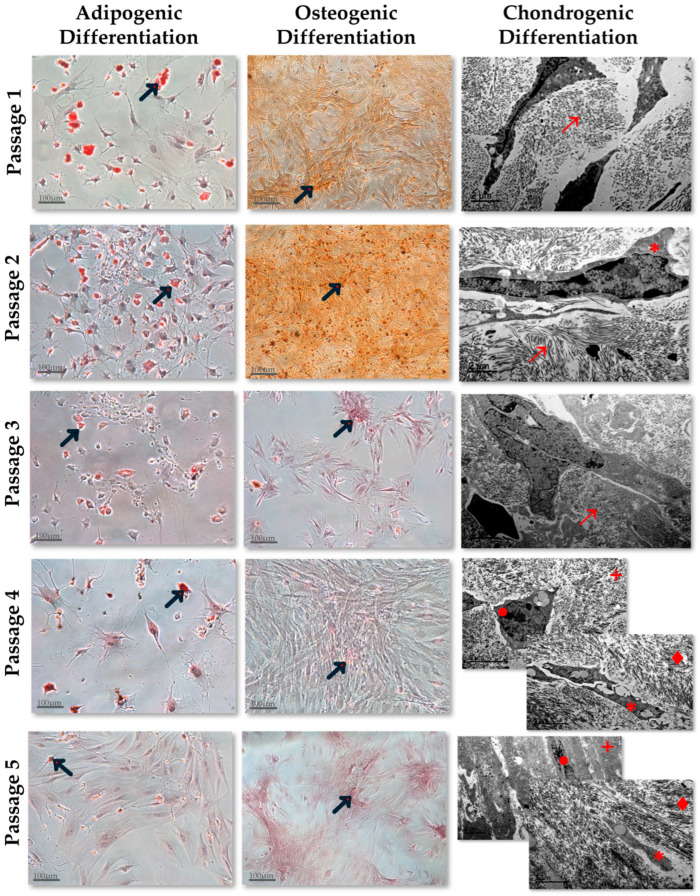
Adipogenic, Osteogenic, and Chodrogenic Differentiation Capacity of Bone Marrow-derived MSCs, in the culture passages 1, 2, 3, 4, and 5. Adipogenic and osteogenic differentiation were analyzed by optical microscopy with a scale bar = 100 µm. The black arrows (→) mark the fat vacuoles in adipogenic differentiation imaging and the Ca+ deposits in the osteogenic differentiation imaging. Chondrogenic differentiation was analyzed by transmission electron micrograph with a scale bar = 2 µm or 5 µm. →: Type III collagen fibers; ∗: Chondroblast; ●: Chondrocyte; +: Matrix; ♦: Cambium.

**Figure 2:**
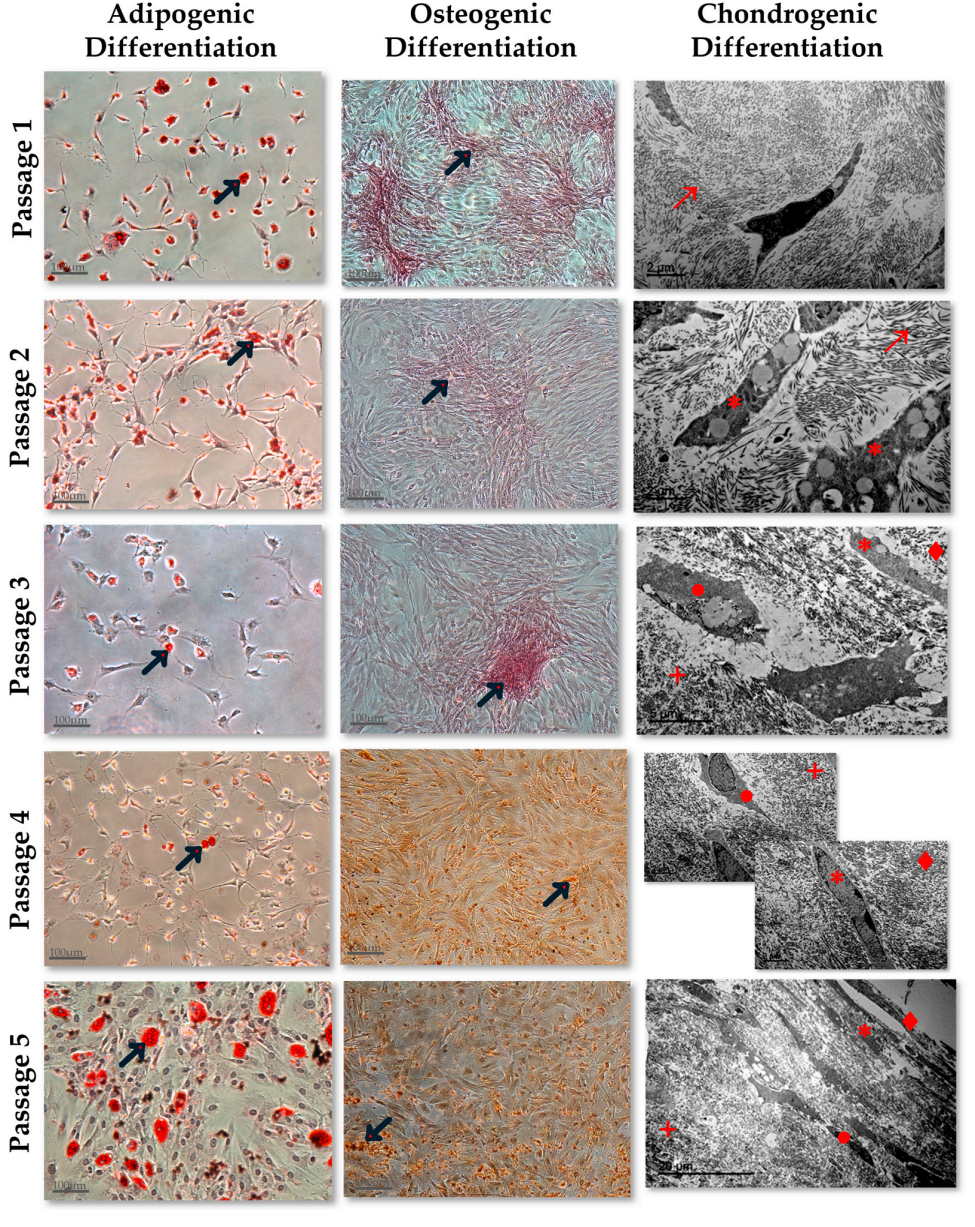
Adipogenic, Osteogenic, and Chodrogenic Differentiation Capacity of Adipose Tissue-derived MSCs, in the culture passages 1, 2, 3, 4, and 5. Adipogenic and osteogenic differentiation were analyzed by optical microscopy with a scale bar = 100 µm. The black arrows (→) mark the fat vacuoles in adipogenic differentiation imaging and the Ca+ deposits in the osteogenic differentiation imaging. Chondrogenic differentiation was analyzed by transmission electron micrograph with a scale bar = 2 µm, 5 µm, or 20 µm. →: Type III collagen fibers; ∗: Chondroblast; ●: Chondrocyte; +: Matrix; ♦: Cambium.

The results obtained in the BM-MSCs and AT-MSCs differentiation assays have been included in Figure 1 and Figure 2, respectively. Regarding the functional characterization of BM-MSCs, their adipogenic differentiation analysis showed that fat vacuole concentrations in cells derived from culture passage 2 were higher than in other culture passages. Practically no fat vacuoles were observed in differentiated cells derived from BM-MSC cultures in passage 5. The results derived from the osteogenic differentiation assay showed that the concentration of positively dyed Ca^+2^ deposits was the highest in BM-MSCs cultured in passage 2 and no deposits were observed in differentiated cells derived from the culture passage 5. Concerning the chondrogenic differentiation, the ultrastructural organization of the differentiated micromasses exhibited an increased differentiation degree as the culture passage progressed. Micromasses derived from BM-MSCs cultured in passages 1, 2, and 3 showed intracytoplasmic tropocollagen as differentiated elements. However, in the micromasses derived from cells cultured in passages 4 and 5, a well-defined organization with differentiated chondroblasts, type III collagen fibers located in the peripheral area (cambium), and chondrocytes-like differentiated in the inner area (matrix) (Figure 1) was observed.

The functional analysis carried out in AT-MSCs expanded in the five culture passages showed that differentiated adipocytes derived from AT-MSCs cultured in passage 5 displayed the highest concentration of fat vacuoles in comparison with the adipocytes derived from the other culture passages. The osteogenic differentiation capacity was also the highest in culture passage 5 in comparison to the other culture passages. The results derived from the chondrogenic differentiation analysis performed exhibited an increased micromass differentiation degree as the culture passage progressed (Figure 2).

### 3.3. Cytokines Quantification in Cell Culture Supernatants

On day seven, the results obtained from VEGF, TGF-β1, and FGF-2 quantifications in BM-MSC supernatants in the first five culture passages did not show statistically significant differences in the comparisons performed between all culture passages (*p* = ns) (see Table 2).

Regarding the quantification carried out in the AT-MSCs supernatants, the concentrations of each cytokine were not statistically different (*p* = ns) in the comparisons performed between the five culture passages. The results are shown in Table 2.

### 3.4. Ultrastructural Analysis by Transmission Electron Microscopy

**Figure 3:**
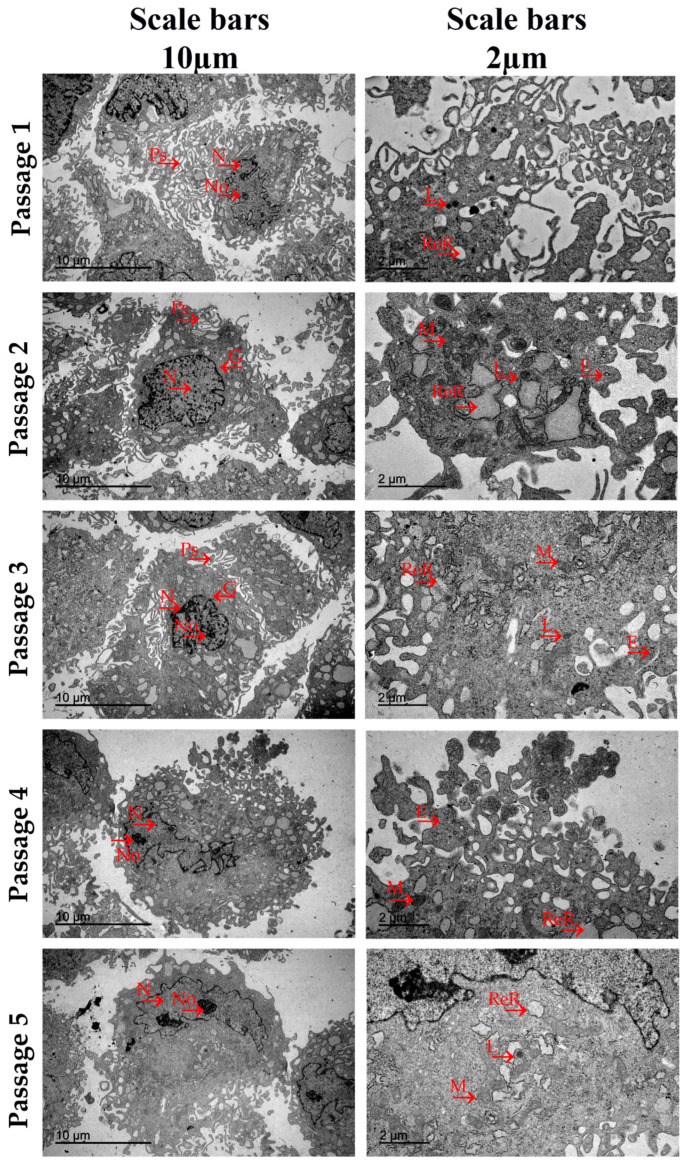
Transmission electron micrograph of bone marrow mesenchymal stromal cells (BM-MSCs) derived from Wistar rats in culture passages 1, 2, 3, 4, and 5. The arrows (→) indicate the main cellular ultrastructures and organelles identified in the BM-MSCs and AT-MSCs in each of their five culture passages. Ps: Pseudopodia. N: Cell Nucleus; No: Nucleolus; ReR: Dilated Cisternae of Rough Endoplasmic Reticulum; M: Mitochondria; G: Cisterns of Golgi; L: Lysosomes; and E: Exosomes. Scale bars = 10 µm and 2 µm.

Results derived from the TEM analysis carried out in BM-MSCs populations expanded in all culture passages have been represented in Figure 3. The TEM analysis showed ultrastructural characteristics that were highly uniform in all the culture passages analyzed. The cell populations cultured in passages 1, 2, and 3 exhibited a greater number of pseudopodia and branched protrusions of the plasmatic membranes. Both elements increased the contact area with the extracellular environment and were indicative of a great adhesion and migration capacity. BM-MSCs in passages 4 and 5 showed a plasma membrane that was more pronounced and regular, as well as fewer protrusions and pseudopodia with a rounded morphology. Large, polymorphic, and eccentric cell nuclei were observed in BM-MSCs derived from all culture passages. These nuclei mainly contained euchromatic, visible nucleoli and deep irregular-shaped notches in their nuclei membrane, morphologic characteristics of high transcription levels of the genetic information into mRNA. Within the rich cytoplasm region, well-developed dilated cisternae of rough endoplasmic reticulum with high proteosynthetic levels, elongated mitochondria, cisterns of Golgi, and lysosomes were present in all the culture passages. The dilated cisternae and mitochondria were present in higher concentrations in the cytoplasm of BM-MSCs expanded in passages 2, 3, and 4 than in cells cultured in passages 1 and 5. Exosomes were observed in BM-MSCs derived from passages 2, 3, and 4 with low electron-dense granules. There were neither fat vacuoles in BM-MSCs expanded in all culture passages nor ultrastructural disruptions. Both observations denoted that cells were not senescent but at an active stage of development.

**Figure 4:**
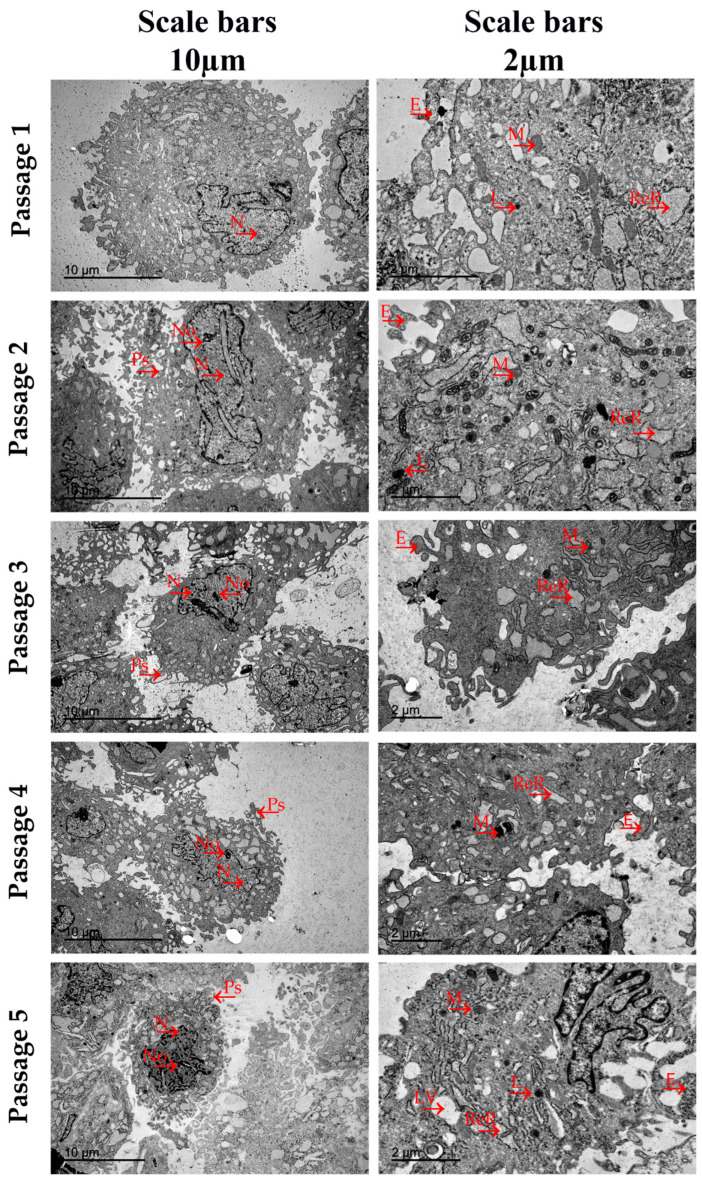
Transmission electron micrograph of AT-MSCs derived from Wistar rats in culture passages 1, 2, 3, 4, and 5. The arrows (→) indicate the main cellular ultrastructures and organelles identified in the BM-MSCs and AT-MSCs in each of their five culture passages. Ps: Pseudopodia. N: Cell Nucleus; No: Nucleolus; ReR: Dilated Cisternae of Rough Endoplasmic Reticulum; M: Mitochondria; L: Lysosomes; E: Exosomes Containing Glycogen; and LV: Lipid Vacuoles. Scale bars = 10 µm and 2 µm.

Results derived from the TEM analysis carried out in AT-MSCs populations expanded in all culture passages have been represented in Figure 4. Regarding MSCs’ ultra morphology, all cells exhibited a rounded morphology with fewer pseudopodia in all the culture passages analyzed. These elements were observed at higher concentrations in AT-MSCs expanded in passages 2 and 3. Large, polymorphic, and lobed nuclei were observed in all culture passages with eccentric positions, visible nucleoli, and higher concentrations of euchromatin for gene transcription. In AT-MSCs cultured in all culture passages, the cytoplasm appeared abundantly rich in organelles, dilated cisternae of rough endoplasmic reticulum with high levels of protein synthesis, large, electron-dense, and well-organized mitochondrial structure, cisterns of Golgi, and lysosomes. Non-disorganized cytoplasm and mitochondria with evident signs of ultrastructural disruption were observed. AT-MSCs derived from passages 1, 2, and 3 exhibited large concentrations of exosomes with electron-dense granules of glycogen. Fat vacuoles were observed in the cytoplasm of AT-MSCs expanded in passage 5, a typical characteristic of fat-origin cells.

## 4. Discussion

In the present study, we have analyzed the functional characteristics and morphology at the ultrastructural level of BM and AT tissue-derived-MSCs isolated from male Wistar rats in their first five culture expansion passages, to identify phenotypic, functional, or ultrastructural differences between the culture passages.

First, we analyzed the immunophenotype and differentiation capacity to characterize each MSC population.

The phenotype was analyzed through the quantification of the MSC-specific markers CD29 and CD90, and the lacked markers CD34 and CD45. These phenotypic criteria to define rat-derived MSC populations had been previously well established through protein expression profiles carried out in many published studies designed to compare their in vitro characteristics [27,28] or to analyze their in vivo functionality [29,30]. Our results demonstrate that MSCs isolated from BM exhibit a typical MSC phenotype in culture passages two, four, and five. In these passages, BM-MSCs express the percentage of the CD45 and CD34 hematopoietic markers as lower than 1% and the percentage of the MSC-specific protein markers CD90 and CD29 as higher than 90%. Regarding culture passages one and three, the protein marker CD90 is the only one for which BM-MSC populations show a percentage slightly lower than 90%. This reduction might not infer the functionality of the global BM-MSC population in both culture passages; however, specific functional studies are needed to confirm this observation to elucidate the intrinsic mechanism related to the CD90 marker in BM-MSC functionality. As to the phenotype of the AT-derived MSCs, this cell population presented a typical MSC phenotype in all culture passages analyzed.

The other important criterion to characterize the different MSC populations is their multipotential differentiation capability into mesodermic lineage cells. We carried out this differentiation potential analysis through the induction of adipocytic, osteoblastic, and chondrocytic differentiation. The adipocytic and osteoblastic differentiation capacities of the differentiated MSCs were identified through the positive Oil-Red and Alizarin-Red staining, respectively, and the chondrocytic differentiation capacity through the differentiation degree and the ultrastructural organization of the differentiated chondrocytic micromasses [19,26]. BM-MSCs exhibit a heterogeneous differentiation profile towards the three mesodermic lineages in the first five culture passages, whereas the differentiation potential profile of AT-MSCs is homogeneous for the three mesodermic lineages. The BM-MSCs’ adipogenic and osteogenic differentiation capacity is higher in early than in later passages and their chondrogenic differentiation capacity is increased as the culture passage progresses. On the other hand, AT-MSCs show a differentiation potential towards the three mesodermic lineages lower in the early passages and higher in the last passages. According to these observed results, we compared the differentiation potential between homologous passages of both MSC types and we found that MSCs derived from BM and AT exhibit similar chondrocytic differentiation profiles but not similar adipocytic and osteoblastic differentiation profiles.

Many published studies have analyzed and compared the immunophenotype and the differentiation capacity of MSCs derived from BM and AT in a single culture passage [18,26]. Some of these concluded that MSCs derived from different sources exhibit differences in their adipogenic and osteogenic differentiation potentials when they compare both cell types in the same culture passage [18,27,28], as we did according to our study-derived observations, although other studies reported similar differentiation capacities in cells derived from bone marrow and adipose tissue both expanded in the same culture passage [22]. These contradictory conclusions observed between the published results could be due to the different culture passages in which MSCs have been analyzed in each study. Regarding the chondrogenic differentiation potential, the results derived from these reviewed studies [18,27,28] conclude that MSCs derived from BM and AT expanded in the same culture passage show no significant differences in their chondrogenic differentiation potential. The observations derived from our chondrogenic analysis agree with these published results.

However, there is not much information published related to the immunophenotype and differentiation potential of both cell types analyzed in different culture passages [21,22,23]. One of these published studies was carried out by Dimitrieva et al. [21], in which they compared the immunophenotype and functionality of both cell types in six culture passages. Kern et al. [22] also performed another comparison study with cells expanded to culture passage seven. Both published reports were developed with human MSCs. Mantovani et al. [23] compared the morphology and functionality of BM-MSCs and AT-MSCs derived from Wistar rats of different ages in successive culture passages. According to our results, these published studies found that MSCs derived from both sources exhibited typical MSC phenotypes in all culture passages analyzed and a different degree of adipocytic and osteoblastic differentiation capacity depending on the analyzed passage. The chondrogenic differentiation capacity was only analyzed in Kern’s study [22] and they observed that MSCs derived from BM and AT were capable of differentiating into a chondrocyte micromass in all culture passages analyzed. According to these results, we observed differentiated chondrocyte micromasses in all culture passages from BM- and AT-derived MSCs. In addition, we analyzed the micromasses’ development degrees with a TEM and we observed that MSCs derived from BM and AT showed similar chondrogenic potential in homologous culture passages. However, we have observed that this differentiation capacity is lower in the early culture passages, in which the micromasses are composed of basic chondrocytic elements that are not well organized. In contrast, in the later culture passages, all micromass elements are well organized with chondroblasts and type III collagen fibers located in the cambium and mature chondrocytes in the matrix. Our TEM analysis allowed us to obtain more details of the micromass differentiation degree at the ultrastructural level, whereas most of the published studies have just been carried out with an identification of the safranin O staining under the microscope light, which provides less information than our analysis.

We consider relevant the differences found in our results derived from the differentiation potential of BM and AT in the different culture passages analyzed. For this reason, and to support our observations, more studies are needed to prove this point and fully elucidate the intrinsic molecular mechanism involved in the MSCs differentiation process.

Once the immunophenotype and the differentiation capacity of BM and AT tissue-derived-MSCs expanded in different culture passages, we carried out the analysis of another functional property of MSCs such as their cytokine secretion capacity.

Previous studies have demonstrated that MSCs release a plethora of angiogenic factors including FGF-2, VEGF, and TGF-β to extracellular space, which promote their angiogenesis ability [31,32]. This angiogenic potential in MSCs derived from different sources has been reported but results derived from these published studies are sometimes conflicting. Thus, Hsiao et al. compared the paracrine potential of AT-MSCs and BM-MSCs and observed that cells derived from AT showed higher levels of cytokine secretion such as VEGF and TGF-β [33] and, paradoxically, another published study found out that BM-MSCs were more proangiogenic than AT-MSCs [34]. Concerning the FGF-2 secretion capacity of MSCs derived from different sources, there are no consistent results in the comparison performed with their secretomes [35]. All these published studies have analyzed and compared the MSCs secretion capacity in a single culture passage.

It is well known that the MSC-derived secretome can be affected by MSC culture conditions, such as the source of the cells, the number of culture passages carried out before their applications, the culture medium and supplementary factors, and modifications in cell handling such as freezing or thawing [36]. However, the lack of standardization and regulation regarding these culture techniques prevents the definition of ideal culturing settings for producing the most effective secretome. In this regard, our work offers a direct comparison of the secretion capacity between MSCs derived from BM and AT and expanded in different culture passages with well-defined culture conditions through the quantification of three angiogenic-related factors (VEGF, FGF-2, and TGF-β). The results derived from this comparison showed non-significant differences in the secretion capacity of these cytokines, so we can conclude that MSCs derived from BM and AT maintain their secretion capacity in the culture passages analyzed.

On the other hand, for the design of this study, we hypothesized that MSC morphology at the ultrastructural level could elucidate some functional properties, such as their paracrine function, through the identification of microvesicles, endosomes, exosomes, and other cytoplasmic organelles which are critical to cellular metabolism and protein synthesis [37]. To prove this, we carried out a TEM analysis of the ultrastructural morphology of MSCs derived from BM and AT expanded in the first five culture passages. Few published studies have analyzed or compared the cell biology of MSCs derived from different sources at the ultrastructural level [38,39,40]. Mantovani et al. [23] performed one of the few comparative studies in this regard. They compared Schwann cells, BM-MSCs, and AT-MSCs from neonatal (neonatal puppies), adult (ten months old), and old Wistar rats (twenty months old), expanded to culture passage seven, and they concluded that MSCs derived from BM and AT isolated from rats with the same age exhibited similar ultrastructural characteristics.

From our TEM study, we can conclude that BM-MSCs and AT-MSCs show a typical mesenchymal ultrastructural morphology [39,40,41] and exhibit morphological characteristics that are relatively uniform in all analyzed culture passages. Both cell types showed large, polymorphic, and eccentric cell nuclei with euchromatic, visible nucleoli and deep irregular-shaped notches in their nuclear membrane. In the cytoplasmic region, they presented well-developed dilated cisternae of rough endoplasmic reticulum (RER), a high concentration of elongated mitochondria with an electron-dense matrix surrounding the RER, cisterns of Golgi, and scarce lysosomes. In the case of BM-MSCs, the concentrations of dilated cisternae and mitochondria were higher in cells expanded in passages two, three, and four in comparison to passages one and five. These different organelle concentrations have not been observed in the different AT-MSC culture passages.

According to the published literature, dilated cisternae and well-organized mitochondrial structures are two cellular characteristics associated with high protein-synthesis levels and cellular energy metabolism, respectively. In addition, mitochondria play an essential role in cell viability and their function is fundamental in processes such as cell differentiation [42], cell proliferation, and aging in many cell types [43]. Moreover, deep irregular-shaped notches in the nuclear membrane and visible euchromatin and nucleoli observed in nuclei morphology are characteristics of cells with high levels of DNA transcription [44]. Taken together, these data might suggest that BM-MSCs expanded in passages two, three, and four could have increased their secretome with respect to passages one and five. This difference has not been observed in our results derived from the analysis of the BM-MSC secretion capacity; however, it is important to note that we have only selected three angiogenic-related factors to analyze, and the MSC secretome is composed of a wide variety of cytokines, chemokines, growth factors, proteins, and extracellular vesicles [45].

Another ultrastructural element that we evaluated in our TEM study was the cell’s plasmatic membrane. Pseudopodia and branched protrusions were observed in a higher concentration in the plasmatic membranes of BM-MSCs and AT-MSCs at early culture passages. However, in the last culture passage, BM-MSCs exhibited higher concentrations of both membrane elements than AT-MSCs, which showed a rounded morphology and fewer protrusions and pseudopodia. It has been widely reported that both membrane elements can increase the contact area with the extracellular environment, and these are related to cell adhesion and migration capacity [46]. In this scenario, we can argue that MSCs in early culture passages could have increased their adhesion ability and migration capacity to chemoattractants.

One of the most important ultrastructural elements observed in our TEM analysis was the exosomes. We have found out that MSCs derived from both sources exhibit higher exosome concentrations in the early passages compared to the last passages. Curiously, the BM-MSC populations exhibit exosomes containing low electron-dense granules, while exosomes generated in AT-MSC populations present high electron-dense granules. Furthermore, these electron-dense granules have been observed at higher concentrations in all analyzed culture passages of the MSCs derived from AT in comparison to BM-MSCs. These electron-dense granules have been previously identified as glycogen [47]. According to the published literature, the proteomic analysis performed on exosomes and proteasomes derived from MSCs showed the frequent appearance of glycolytic enzymes, which play an important role in the maintenance of cellular functionality [47]. In this scenario, we can suggest that AT-MSCs can maintain a more stable cellular functionality throughout the different culture passages analyzed in comparison to MSCs derived from BM. In this scenario, we can argue that MSCs in early culture passages could have increased their adhesion ability and migration capacity to chemoattractants.

## 5. Conclusions

Considered as a whole, and summarizing the results from the analysis carried out to the immunophenotype, differentiation ability, cytokine secretion capacity, and ultrastructural morphology of BM and AT tissue-derived-MSCs expanded in different culture passages, we can conclude that these results evidence that both cell types display characteristics that are different depending on their culture passages, so the number of the culture passage must be considered according to the MSCs’ clinical or preclinical applications.

## Figures and Tables

**Table 1:** Phenotypic quantification by flow cytometry: phenotypic characterization of bone marrow-derived mesenchymal stromal cells (BMMSCs) and adipose tissue-derived mesenchymal stromal cells (ATMSCs) isolated from Wistar rats and expanded in culture passages 1, 2, 3, 4, and 5. Data are represented as mean ± SD from each experimental group. *p*-value = ns.

Culture Passage	Cell Type	%CD29	%CD90	%CD34	%CD45	Side Scatter	Forward Scatter
1	AT-MSCs	98.91 ± 1.23	93.27 ± 6.15	0.05 ± 0.04	0.08 ± 0.09	455.1 ± 36.8	476.4 ± 58.2
	BM-MSCs	92.82 ± 8.17	87.81 ± 12.31	0.32 ± 0.35	0.37 ± 0.34	418.99 ± 69.79	459.0 ± 110.0
2	AT-MSCs	99.06 ± 0.85	95.94 ± 3.03	0.04 ± 0.03	0.07 ± 0.06	433.3 ± 213.9	514.2 ± 79.1
	BM-MSCs	98.04 ± 1.58	97.44 ± 2.46	0.018 ± 0.04	0.31 ± 0.09	596.1 ± 153.5	521.1 ± 45.3
3	AT-MSCs	98.71 ± 1.29	96.79 ± 6.09	0.03 ± 0.03	0.06 ± 0.06	461.3 ± 57.5	541.5 ± 35.1
	BM-MSCs	94.38 ± 5.01	80.62 ± 15.79	0.09 ± 0.09	0.37 ± 0.21	628.9 ± 229.5	367.7 ± 17.1
4	AT-MSCs	99.24 ± 0.60	98.87 ± 0.71	0.03 ± 0.02	0.05 ± 0.04	456.3 ± 35.5	589.09 ± 33.96
	BM-MSCs	96.57 ± 2.24	90.14 ± 7.99	0.26 ± 0.03	0.39 ± 0.09	698.7 ± 3.5	412.3 ± 18.1
5	AT-MSCs	98.46 ± 1.11	97.56 ± 1.96	0.02 ± 0.02	0.05 ± 0.04	558.0 ± 54.8	622.7 ± 72.9
	BM-MSCs	97.37 ± 2.50	95.40 ± 4.38	0.12 ± 0.09	0.41 ± 0.02	773.0 ± 44.9	548.1 ± 37.4

**Table 2:** Cytokine quantification in culture supernatant from bone marrow-derived mesenchymal stromal cells (BM-MSCs) and adipose tissue-derived mesenchymal stromal cells (AT-MSCs) in five culture passages (P1, P2, P3, P4, P5). VEGF, TGF-β1, and FGF-2 concentration (pg/mL) have been expressed per 10,000 cultured cells in 24 h. Data are represented as mean ± SD from each experimental group. *p* Value = ns.

	VEGF (pg/mL)		TGF-β1 (pg/mL)		FGF-2 (pg/mL)	
	BM-MSCs	AT-MSCs	BM-MSCs	AT-MSCs	BM-MSCs	AT-MSCs
P1	7.32 ± 0.25	7.49 ± 0.04	33.89 ± 1.49	18.69 ± 0.07	2.78 ± 0.17	1.32 ± 0.01
P2	6.76 ± 0.01	9.12 ± 0.01	29.98 ± 0.58	26.39 ± 0.13	3.01 ± 0.13	2.81 ± 0.01
P3	13.61 ± 0.001	17.08 ± 0.03	49.74 ± 0.07	33.69 ± 0.24	6.21 ± 0.01	5.71 ± 0.02
P4	10.22 ± 0.32	20.06 ± 0.22	47.23 ± 0.60	49.74 ± 1.02	6.49 ± 0.21	8.17 ± 0.04
P5	7.38 ± 0.61	24.72 ± 0.04	41.38 ± 3.30	71.94 ± 0.20	7.61 ± 0.84	15.42 ± 0.04

## Data Availability

Data is contained within the article.
